# Perception of the Level of Competency of Candidates for Graduation: A Multidisciplinary Approach to Complex Thinking

**DOI:** 10.3390/jintelligence11100202

**Published:** 2023-10-20

**Authors:** José Carlos Vázquez-Parra, Marco Cruz-Sandoval, Paloma Suárez-Brito

**Affiliations:** 1 Institute for the Future of Education, Tecnologico de Monterrey, Monterrey 64849, Mexico; 2 Center for the Future of Cities, Tecnologico de Monterrey, Monterrey 64849, Mexico

**Keywords:** professional education, educational innovation, future of education, complex thinking, Latin American education, higher education

## Abstract

Complex thinking is a cognitive skill that focuses on the integrated analysis and synthesis of information with a systemic and critical perspective that enables creative decision-making in the face of complex realities or challenges. At the educational level, it is valued as a transdisciplinary competency, meaning it is relevant for individuals regardless of their profession or field of study. This article presents the results of measuring the perceived achievement of complex thinking among 830 graduating students from a technological university in Mexico, aiming to identify possible significant differences based on their discipline or major. Methodologically, a multivariate descriptive statistical analysis was performed using R and RStudio software, including calculation of means and standard deviations, violin plots, boxplot and ANOVA significance analysis, and *t*-test. The results show that the differences were not statistically significant in all the disciplines, although it is possible to note significant differences, which reveals a differentiated behavior in the process of formation and development of complex thinking according to the discipline of study. In conclusion, the present study shows that the students’ areas of training are associated with differences in perception of complex thinking and its associated sub-competencies, thus differentiating this ability in their graduation profile. This article contributes to the existing literature on the formation and development of complex thinking and its sub-competencies as relevant professional skills for lifelong learning.

## 1. Introduction

Complex thinking is the cognitive ability to understand reality integrally, recognizing the connections between different elements and the unpredictability and uncertainty that characterize the current world (Tobón and Luna 2021). It stands on a global multidisciplinary approach that seeks to avoid simplifications and reductionism by addressing issues broadly and holistically (Vertovec 2023). At the professional level, the importance of this skill is acknowledged for tackling challenges in society, industry, and contemporary work, making it a competency applicable in all disciplines (Cheon et al. 2019). In this regard, complex thinking becomes increasingly important in university education, considering that any future professional, regardless of their background, must confront problems that transcend a single discipline, requiring more comprehensive skills employing multiple viewpoints to comprehend and solve complex challenges (Baena et al. 2022).

At the professional level, the challenges faced by contemporary organizations are typically interconnected, uncertain, and dynamic. Therefore, to address such issues, it is essential to have personnel with sufficient and relative cognitive abilities (Hiver et al. 2021).

Based on the above, the objective of this article is to present the results of measuring graduating students’ perceived achievement of complex thinking competency and its sub-competencies at a technological university in Mexico. This sample includes students from the six disciplines offered by this institution: Humanities and Education, Social Sciences and Government, Engineering, Health Sciences, Business, and Architecture and Design. This study aimed to identify the level of competency perceived by students at the end of their educational process, identify possible disciplinary and career differences, and lay the groundwork for future studies on the relevance of promoting complex thinking as a transversal competency applicable to graduates’ profiles in any educational institution. To achieve this, a multivariate descriptive statistical analysis was performed using R (4.2.2 version) and RStudio (2023.6.0.421 version) software, including calculation of means and standard deviations, violin plots, boxplot and ANOVA significance analysis, and *t*-test.

### 1.1. Theoretical Framework

#### 1.1.1. Complex Thinking as a Transdisciplinary Competency

There is no doubt that a characteristic of the current world, especially after the COVID-19 pandemic, is the uncertainty and volatility of social and cultural systems, which comprise multiple interconnected elements interacting in unpredictable ways (Alkhatib 2019). Thus, a complex world requires sufficient relevant skills, making complex thinking an ideal competency.

It is important to note that there is no agreed definition of what complex thinking is; however, studies on this competence tend to adhere to certain academically established visions (Melo et al. 2020), which for this specific research will be the approach of the French sociologist and philosopher Edgar Morin. According to Morin (1990), complex thinking is a cognitive ability developed by individuals that allows them to understand reality with its inherent complexity, avoiding the simplification and reductionism of systems and phenomena.

Unlike more traditional approaches, complex thinking is holistic and integrative, analyzing phenomena not in isolation but based on understanding relationships, interactions, and patterns within complex systems (Horn et al. 2022). It allows for recognizing the uncertainty and ambiguity of reality, employing multiple perspectives, and tolerating constantly changing environments.

Being a high-level cognitive competency, complex thinking comprises other sub-competencies that provide versatility and adaptability in approaching the environment, such as systemic thinking, scientific thinking, critical thinking, and innovative thinking (Vázquez-Parra et al. 2022). These sub-competencies allow individuals to develop an integrated view of reality, enabling them to address challenges in their personal and professional spheres (Cruz-Sandoval et al. 2023b).

Specifically, systemic thinking allows for a global understanding of phenomena, recognizing connections between elements and their interactions (Jaaron and Backhouse 2018). Scientific thinking uses objective and validated methods to solve problems, emphasizing the importance of evidence (Koerber and Osterhaus 2019). Critical thinking involves questioning and reevaluating existing knowledge to address current needs and assessing and enriching information (Cui et al. 2021). Finally, innovative thinking goes beyond critical thinking to include cognitive and operational aspects and personal variables like motivation and flexibility to generate socially and culturally accepted new products and solutions (Saienko et al. 2021). While each sub-competency can be acquired and developed in isolation for their cognitive value, the integrated exercise of all four achieves a genuinely integrated vision of approaching complex problems (Ramírez et al. 2021).

Logically, this holistic, systemic, and broad perspective of reality makes complex thinking transdisciplinary, meaning it can be applied to address problems and challenges that transcend the boundaries of a single discipline (Drucker 2021). Many issues within work environments tend to break disciplinary barriers, requiring diverse viewpoints and the cognitive elements characteristic of various academic areas (Sherblom 2017). In this sense, complex thinking integrates different disciplinary approaches, contributing to a more relevant professional vision that aligns with the current needs of industries and professions. From a pedagogical perspective of challenge-based learning, training in complex thinking brings a wide range of advantages, as it enables students to develop skills to recognize interconnections, work collaboratively, integrate multiple perspectives and visions into their decision-making process, and develop a more comprehensive and profound understanding of reality (Silva 2020).

In addition to this, there are other benefits of complex thinking training, as reported by several authors (Zhou 2021; Vázquez-Parra et al. 2022; Castillo-Martínez et al. 2022):

- The ability to understand problems from various angles for action, providing more robust, well-argued, and innovative solutions;
- The ability to adapt and be flexible in the face of constant changes in industry, market evolution, and professional needs;
- A broad vision of the causes and roots of problems and the potential consequences of actions, considering elements that may be overlooked in linear thinking;
- Development of an innovative and creative perspective, facilitating new routes for solving identified issues;
- Promoting multidisciplinary collaboration, fostering teamwork, and recognizing the richness of diverse knowledge and perspectives.

In this regard, the relevance of complex thinking training is irrespective of the career or discipline of education, as the skills provided by this cognitive competency are valuable for any professional (Vázquez-Parra et al. 2023a, 2023b). Universities must undertake specific actions to identify how complex thinking is acquired and developed in their curricula. This study provides a valuable approach to understanding how to adopt this competency in the graduation profiles of students from different disciplines.

#### 1.1.2. State of the Art

Based on the above, the reflection of complex thinking as a pedagogical competence has been considerably addressed by previous studies, although focusing on concrete elements such as certain disciplines or its intersection with social or behavioral factors of students and teachers.

Antonio et al. (2004) present the effects of racial diversity on the development of complex thinking in a group of students from three universities, discussing how social context and interactions in the face of racial diversity influence the level of development of complex thinking. At the teaching level, Zepke (2011) reports a study in which he seeks to understand how complex thinking can influence not only students’ engagement with their subjects but also their teachers’ motivation. Meanwhile, Forsman et al. (2014) studied the level of complex thinking in university students in the disciplines of physics and engineering, analyzing how having a high level of complex thinking can influence the retention level of students, providing tools to view and analyze complex educational issues in higher education in terms of nested, interdependent and interconnected systems. Tobón and Luna (2021) link the development of complex thinking competence with training in sustainable social development, considering that the cognitive skills developed by a complex thinker are very appropriate for teaching contemporary social challenges.

It is important to note that the present study arises as a result of the research work of a research group dedicated to the study of complex thinking, and therefore, there are relevant antecedents regarding this cognitive competence. It is possible to mention some previous studies that made a measurement similar to the one proposed in this article but that did not consider students who were candidates to graduate, which made the results not accurate because they did not consider the closure of the university process (Vázquez-Parra et al. 2022). There are also other studies that have paid more attention to the gender element, certain sub-competencies associated with some specific disciplines, and even relevant social factors in the Latin American context (Cruz-Sandoval et al. 2023a; Carlos-Arroyo et al. 2023).

The intention of making these remarks is to highlight the originality and value of the present study, as it is the first reflection that considers such a large group of graduating students from all disciplines.

## 2. Materials and Methods

A random convenience sample of 830 students (455 Men and 377 Women) in the last semester of all the disciplines offered by the institution (Humanities and Education, Social Sciences and Government, Business, Engineering, Architecture and Design, and Health Sciences) included data collected during 20–24 April 2023, from an orientation course for graduate candidates organized by the Campus Life and Professional Development Center. The students responded voluntarily to a self-administered questionnaire digitalized on the Google Forms platform. Table 1 shows more information regarding the characteristics of the sample.

Although the population was not proportional among the disciplines, it did correspond to the characteristics of the institution’s student body. Being a technological university, most careers in the sample population corresponded to engineering. Also, this institution has gained recognition for its business training, which shows in the number of sample participants in this discipline. The other areas correspond to the number of students in their majors.

### 2.1. Ethical Considerations

Since this exploratory study involves human beings, its implementation was supervised and approved by the interdisciplinary research group R4C, and it had the technical support of the Writing Lab of the Institute for the Future of Education of Tecnologico de Monterrey.

As part of this supervision, the personal information that the present study could use was regulated, considering that, for the objectives of this research, it was sufficient to have access to the results of the questionnaire, the discipline, the study career, and the gender of the participants. For this reason, there is no further information associated with the sample. Additionally, it was determined that the implementation process of the instrument would be self-administered, sending a link to the questionnaire to the participants’ homes. The implementation process was only for the collection of information related to this instrument, i.e., it did not consider additional questions or information. In order to answer the instrument and access the items, the individual had to accept the privacy notice and the terms and conditions assigned by the institution for this type of research. This ensured that all participants expressed their consent and prevented their responses from being influenced by any implementer.

### 2.2. Instrument and Data Analysis

This study employed the validated eComplexity instrument to measure participants’ perception of their mastery of complex thinking and its sub-competencies. The eComplexity instrument has the objective of measuring the level of perception of achievement that participants have of the complex thinking competency and its sub-competencies. It is an instrument that has been validated both theoretically and statistically, as well as by a team of experts in the field (Castillo-Martínez et al. 2022). As for the structure of the instrument, it is made up of 25 items divided into three sub-competencies, systemic thinking, scientific thinking, and critical thinking, and each of these three sub-competencies was, in turn, divided into three areas: knowledge, skills, and attitudes or values. Although the instrument was shown to have high validity and reliability, after statistical analysis, modifications were made considering the observations of the experts. The improved version was validated with 443 participants who evidenced the reliability with the internal consistency of the instrument for complexity reasoning competence (Ramírez et al. 2021). The validation procedure of the eComplexity instrument consisted of a first phase of theoretical validation, as well as an expert validation was performed to validate the content of the instrument. Based on the theoretical and content validation by means of expert judgment, it was determined that the eComplexity instrument is highly valid and reliable (Castillo-Martínez et al. 2022). The items are a set of statements related to characteristics associated with their sub-competencies:

1. I have the ability to find associations between variables, conditions, and constraints in a project;
2. I identify data from my discipline and other areas that contribute to solving problems;
3. I participate in projects that need to be solved using inter/multidisciplinary perspectives;
4. I organize information to solve problems;
5. I enjoy learning different perspectives on a problem;
6. I am inclined to use strategies to understand the parts and the whole of a problem;
7. I have the ability to identify the essential components of a problem to formulate a research question;
8. I know the structure and formats for research reports used in my area or discipline;
9. I identify the structure of a research paper used in my area or discipline;
10. I apply the appropriate analysis methodology to solve a research problem;
11. I design research instruments consistent with the research method used;
12. I formulate and test research hypotheses;
13. I am inclined to use scientific data to analyze research problems;
14. I have the ability to critically analyze problems from different perspectives;
15. I identify the rationale for my own and others’ judgments to recognize false arguments;
16. I self-evaluate the level of progress and achievement of my goals to make the necessary adjustments;
17. I use reasoning based on scientific knowledge to make judgments about a problem;
18. I make sure to review the ethical guidelines of the projects in which I participate;
19. I appreciate criticism in the development of projects in order to improve them;
20. I know the criteria for determining a problem;
21. I have the ability to identify variables from various disciplines that can help answer questions;
22. I apply innovative solutions to diverse problems;
23. I solve problems by interpreting data from different disciplines;
24. I analyze research problems, contemplating the context to create solutions;
25. I tend to evaluate, with a critical and innovative sense, the solutions derived from a problem.

The analysis of the eComplexity instrument considers the evaluation of both the general competency (complex thinking) and each of its sub-competencies (systemic, scientific, innovative, and critical thinking). In this sense, the general competency includes all the items, while in the case of the sub-competencies, these consider specific items: systemic thinking (1–6), scientific thinking (7–13), critical thinking (14–19), and innovative thinking (20–25).

To analyze the data, we used a multivariate descriptive statistical approach through the R software (R Core Team 2017), version 4.2.2, and Rstudio (RStudio Team 2022), version 2023.6.0.42. This analysis included calculating mean values and standard deviations to describe the distribution and variability of the dataset. The mean and standard deviation are basic descriptive statistics that offer valuable insight into the centrality and dispersion of the data. These metrics are the first approach that provides a comprehensive understanding of the data distribution. The mean value, representing the center of the distribution, allowed for finding the balance point of the data, while the standard deviation measured the variability from the mean value. Additionally, we produced violin plots to visualize the density of the data distribution, a graphical tool introduced by Hintze and Nelson (1998). This plot is a visual instrument that combines the features of a Boxplot with a Kernel Density Distribution. One of its primary strengths is that it not only illustrates the central tendency and spread of the data but also its probability density. This aids in pinpointing where the data clusters and where observations are infrequent. It is an essential tool in descriptive analyses, especially when the objective is to comprehend the core structure of the data and the nature of its distribution. Subsequently, significance analyses tested differences in mean values through Analysis of Variance (ANOVA) and *t*-tests. ANOVA is a statistical approach designed to compare the means of three or more independent groups, identifying if there are statistically significant differences between them. One of its primary strengths is its ability to assess multiple groups concurrently, thereby reducing the risk of committing a Type I error, also known as an alpha error or false positive (i.e., when a true null hypothesis is incorrectly rejected). This analysis is crucial in studies that seek to elucidate the effects of categorical variables with three or more categories on a continuous outcome variable. On the other hand, the *t*-test or Student’s *t*-test is a statistical tool used to determine if there are significant differences between the means of two groups. These tests were performed with a 95% confidence interval, employing a *p*-value of 0.05.

## 3. Results

Table 2 presents the results of students’ perceived achievement of complex thinking and its sub-competencies, broken down by disciplines. The data reveal that students in Health Sciences had the highest perception of the overall competency (complex thinking; mean 4.50), while students in Architecture, Art, and Design demonstrated the lowest mean (4.22). Regarding the sub-competency of systemic thinking, students in the School of Humanities and Education perceived themselves with the highest development (4.54), whereas students in the School of Architecture, Art, and Design showed the lowest mean (4.06). Notably, students in the School of Health Sciences perceived the highest development in scientific thinking (4.44), critical thinking (4.56), and innovative thinking (4.52). On the other hand, students in the School of Humanities and Education had the lowest perceived achievement in the sub-competency of scientific thinking (4.12). Regarding critical thinking, students in the School of Social Sciences and Government had the lowest mean in perceived achievement (4.35). Finally, in innovative thinking, students in Architecture, Art, and Design perceived themselves with the lowest achievement (4.25).

**Figure 1:**
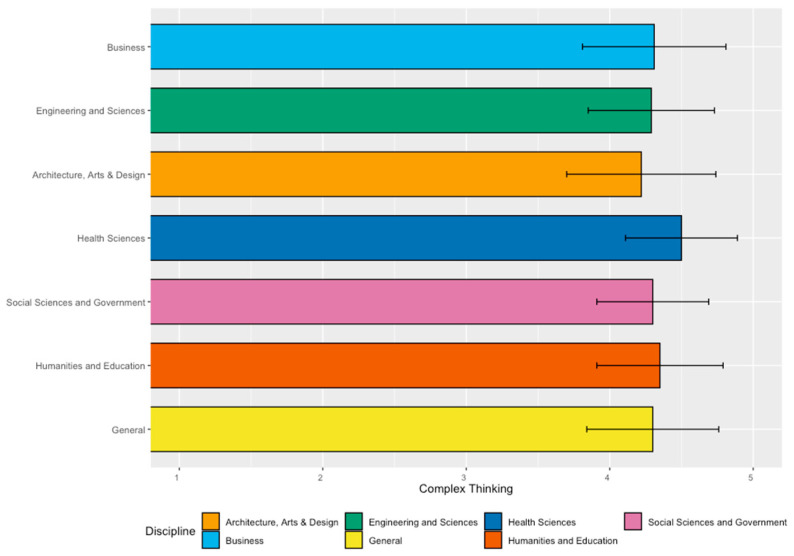
Complex thinking bar graph: mean values and standard deviations by discipline.

Figure 1 presents the students’ perceived achievement of the complex thinking competency. The results show that students in the School of Health Sciences have a significantly higher perception of achievement in this competency than students from other disciplines.

**Figure 2:**
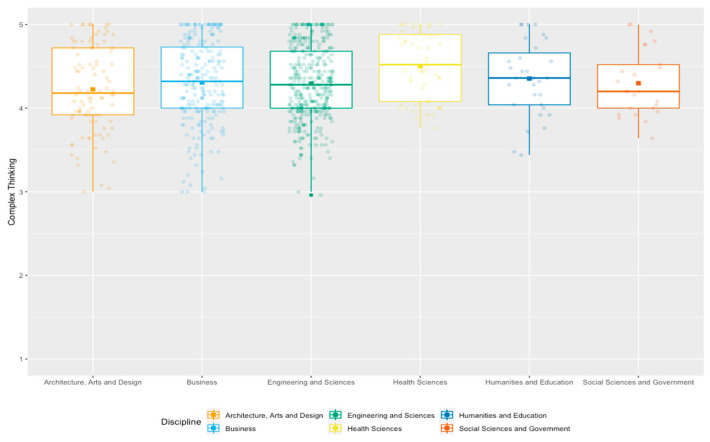
Complex thinking boxplot analysis by discipline.

Figure 2 is a boxplot analysis comparing the students’ perception of complex thinking per discipline and the data dispersion. The analysis reveals that, although the mean values are relatively similar between the disciplines, the least dispersion occurs in the Social Sciences and Government, Humanities and Education, and Health Sciences schools. In contrast, the Architecture, Sciences and Engineering, and Business students had a wider data dispersion in the mean values of perceived complex thinking.

**Figure 3:**
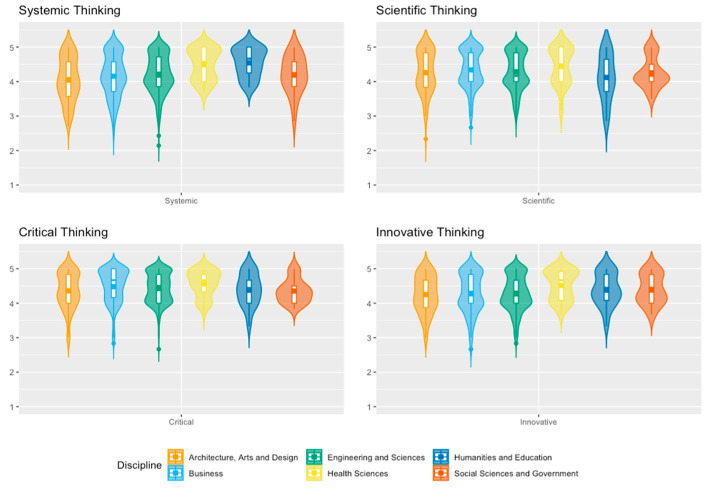
Sub-competencies of complex thinking: violin plots of sub-competencies by discipline.

Figure 3 shows the analysis of the distribution of students according to their perception of different sub-competencies of complex thinking in their area of study. Students in Health Sciences and Humanities and Education had a higher concentration of high values in the perceived achievement of systemic thinking. In contrast, students in the Engineering and Sciences and Business schools had a distribution with lower values in this sub-competency. Notably, the Humanities and Education and Architecture schools had the highest distribution of low perceived achievement values in scientific thinking. Regarding critical thinking, there is a notable high distribution of values of high perceived achievement among the School of Health Sciences students. Similarly, in innovative thinking, the School of Health Sciences also presented a high distribution of students with an elevated perception of this skill.

Table 3 presents the results of the ANOVA analysis of the mean values of students’ perceived achievement in the competency of complex thinking and its sub-competencies. The analysis aimed to determine if significant statistical differences existed between the disciplines in students’ perceived achievement. We used a significance level of *p* < 0.05. The results showed that there were indeed significant differences between disciplines in the sub-competencies of systemic thinking and innovative thinking, as well as the overall competency of complex thinking.

Table 4 analyzes the perceived achievement of complex thinking in different areas of the School of Engineering and Sciences. Note that students who showed a higher perception of development in complex thinking majored in Digital Transformation (4.66), Data Science (4.56), and Electronic Technologies (4.51). In contrast, the Robotics and Digital Systems majors had the lowest perception of development in this competency (3.88).

**Figure 4:**
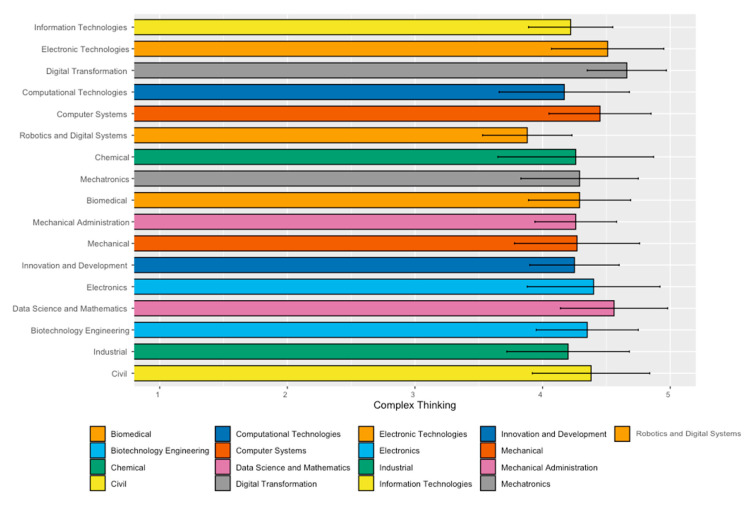
Complex thinking bar graphic. Analysis of means and standard deviations by career in the School of Engineering and Sciences.

Figure 4 shows the students’ perceived achievement of complex thinking in different careers in the School of Engineering and Sciences, showing the mean values and their respective standard deviations.

Table 5 presents the ANOVA analysis to evaluate significant differences in the mean values of perceived achievement in complex thinking by students in different majors in the School of Engineering and Sciences. The analysis results indicate no significant differences (*p*-value of 0.14) between the careers regarding the perception of achievement in this competency.

Table 6 shows the analysis of students’ perceived achievement of complex thinking in the School of Business. The results highlight that the students in the Business Administration career had the highest perception of achievement (4.50), while the students in Business Intelligence perceived the least development (4.07).

**Figure 5:**
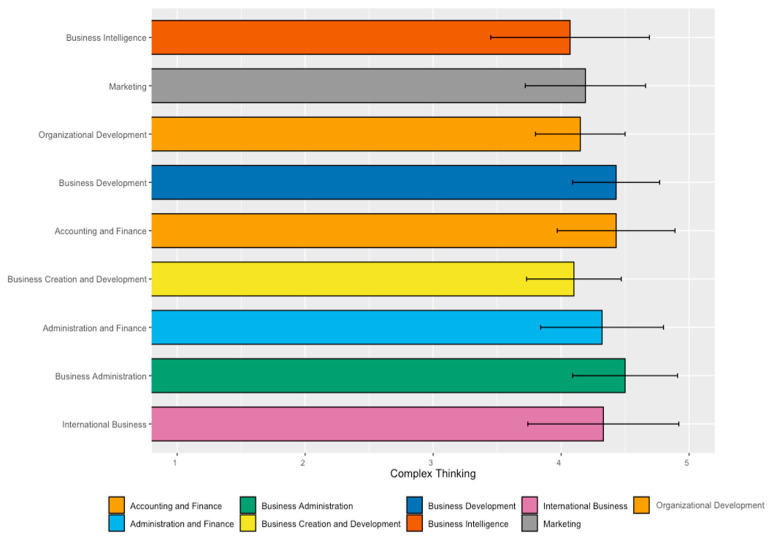
Complex thinking bar graph: analysis of means and standard deviations by major in the School of Business.

Figure 5 presents the mean values and standard deviations of the perception of achievement in the complex thinking competency among students in the School of Business, broken down by major. Note that Business Administration, Accounting and Finance, and Business Development careers had the highest average values in perceived development of complex thinking.

Table 7 analyzes significant differences in the means of the complex thinking competency among students in different careers at the School of Business. An analysis of variance (ANOVA) examined these differences, with results indicating significant differences in perceived achievement among the students attending this school (*p*-value of 0.01).

Regarding the careers of the School of Social Sciences and Government, Table 8 reveals that International Relations students had the highest perceived achievement of complex thinking (4.36) compared to Law students (4.22).

**Figure 6:**
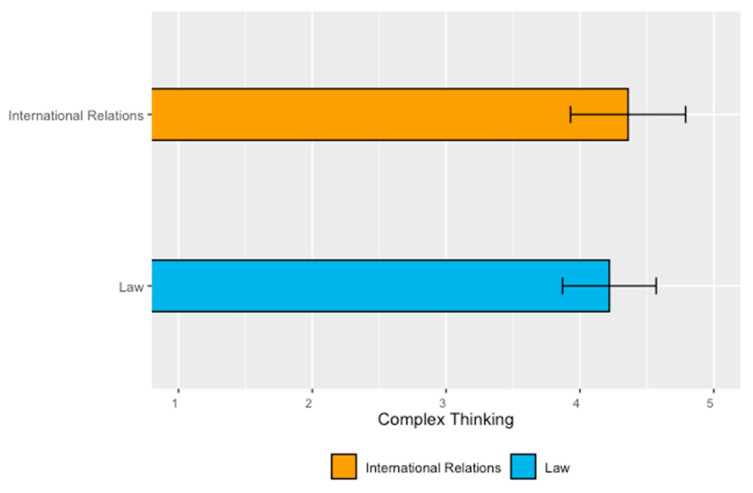
Complex thinking bar graph: analysis of means and standard deviations per career in the School of Social Sciences and Government.

Figure 6 shows the mean values and standard deviations of perceived complex thinking competency among students with Social Sciences and Government majors. The International Relations students had the highest perceived achievement (4.36) and the most variability (SD 0.43).

Table 9 shows the *t*-test on the means of the students’ perceived achievement in the two careers of the School of Social Sciences and Government. The results indicate no significant differences in the perception of this competency (*p*-value of 0.46).

Table 10 presents the analysis of mean values and standard deviations on the perceived achievement of complex thinking by students of the School of Architecture, Art, and Design. The results indicate that Architecture students had the highest perception (4.31), while Digital Art students had the least (4.07).

**Figure 7:**
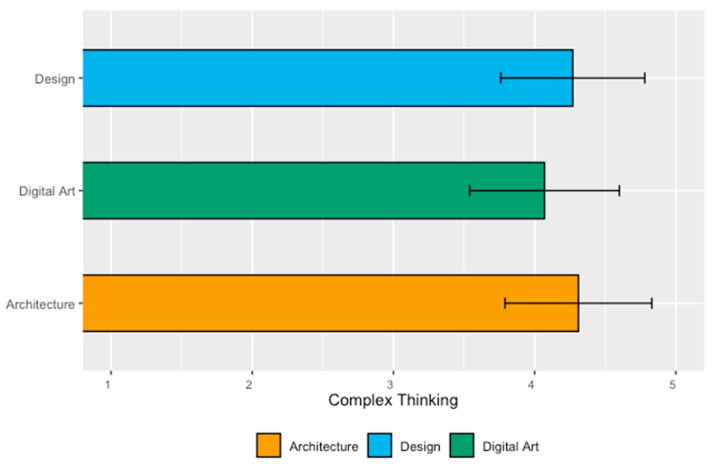
Complex thinking bar graph: analysis of means and standard deviations per career in the School of Architecture, Art, and Design.

Figure 7 complements the results of the previous table. Although the students in the Design major showed a slightly lower perception of complex thinking (4.27) compared to the Architecture students, they had the lowest standard deviation (0.51).

Finally, Table 11 presents the analysis of significant differences in mean values of students in the Architecture, Art, and Design careers concerning the development of complex thinking. The ANOVA analysis shows no significant difference in competency development between the students in the three majors (*p*-value of 0.19).

These analyses were not done for the Humanities and Education and Health Sciences disciplines because their samples reflected only one professional career.

## 4. Discussion and Conclusions

This article presented the measurement of graduating students’ perceived achievement of the competency of complex thinking and its sub-competencies in a technological university in Mexico. The sample included students from the six disciplines offered by this institution: Humanities and Education, Social Sciences and Government, Engineering, Health Sciences, Business, and Architecture and Design. The objective was to identify the level of competency perceived by students at the end of their educational process, describe the data behavior of their responses, and identify possible differences between disciplines and majors.

This report highlights nine relevant findings:

1. Students in Health Sciences showed the highest perceived achievement in the overall competency (complex thinking with a mean value of 4.50), while students in Architecture, Art, and Design had the lowest mean (4.22);
2. Regarding the sub-competency of systemic thinking, students in the School of Humanities and Education perceived themselves with the highest achievement (4.54), while students in the School of Architecture, Art, and Design had the lowest mean (4.06). This could be associated with the fact that the training provided by the humanities tends to focus on the identification and analysis of environmental problems, which could influence the students’ greater awareness of systems and their interconnection. However, this assumption would imply that Social Sciences should also yield similar results, which is not the case. In this sense, there is a need for a specific study on this sub-competence to analyze what particular factors could be influencing students’ perception of certain disciplines;
3. Regarding scientific, critical, and innovative thinking, the School of Health Sciences students had the highest mean. It is interesting to note that Health Sciences students are more likely to be trained within the standard positivist scientific paradigm. The influence of this paradigm could be expressed as an overconfidence in scientific skills, which could lead to students developing a better perception of themselves in this sub-competency. This assumption invites the possibility of developing complementary research, in which the perception of health science students can be explored in a concrete way, seeking not only to identify but also to describe the reason for these high results;
4. Students in the School of Humanities and Education showed the lowest perceived achievement of scientific thinking (4.12), students in the School of Social Sciences and Government had the lowest mean (4.35) of critical thinking, and students in Architecture, Art, and Design had the lowest value in innovative thinking (4.25). It is interesting to pay attention to the results for social sciences and architecture, art, and design since their lowest values are in sub-competencies that tend to be more associated with their discipline. One explanation that could be put forward is that since these are cognitive elements more specific to their area of knowledge (critical thinking for social sciences and innovative thinking for architecture, art, and design), their students are more critical and demanding about what they think they know and about the skills they have. It will be interesting to consider further research to explore these results;
5. The study confirmed a statistically significant difference in the results among disciplines for the overall competency and the sub-competencies of systemic thinking and innovative thinking;
6. In the School of Engineering and Sciences, the best perception of complex thinking was in the majors of Digital Transformation (4.66), Data Science (4.56), and Electronic Technologies (4.51). In contrast, Robotics and Digital Systems students perceived the lowest development in this competency (3.88);
7. In the School of Business, the results highlight that Business Administration students show the highest perception of achievement (4.50), while students in Business Intelligence perceived the least development (4.07). On this particular point, it is important to note that the business intelligence career is relatively new and that the participants in this survey are part of the first generation. In this sense, it is understandable that they are more critical with respect to the way they perceive their skills, this being a perception of the way they conceive that this career prepares them for professional challenges and challenges;
8. Regarding the majors in the School of Social Sciences and Government, students in International Relations had the highest perception of complex thinking (4.36) compared to students in Law (4.22). However, the difference was not statistically significant;
9. Finally, regarding the School of Architecture, Art, and Design majors, the results indicate that students in Architecture had the highest development (4.31), while students in Digital Arts perceived themselves with the least (4.07).

Based on these results, it is possible to conclude that, although the differences were not statistically significant in all cases, noticeable differences can be observed, which highlight a differentiated behavior in the perception of the development of complex thinking per the field of study.

### 4.1. Implications and Future Studies

This research indicates an association between the student’s field of study and their perception of the development of complex thinking and its related sub-competencies. Distinct patterns emerge across disciplines, suggesting variations in how students perceive and develop these competencies upon graduation. As mentioned in the theoretical framework, the competency of complex thinking is highly valued by the job market and contemporary industry, so these results may have implications regarding the opportunities for future graduates and how they face the challenges of their profession. Every educational institution should seek not only the development of competencies and skills but also ensure that this process impacts all students as equitably as possible and guarantee that their processes are as standardized as possible.

In this regard, this study opens the possibility for new research that focuses on each discipline to determine more precisely the process of developing complex thinking in each major. Further studies should not only measure the achieved level but also the improvement through longitudinal measurements that begin when students enter the university and follow until they graduate.

At a practical level, this study provides valuable information for educational institutions interested in developing complex thinking, setting a precedent for those disciplines with deficient competency development that should be strengthened. Furthermore, by identifying disciplines with better results, analyzing their pedagogical practices could help other areas achieve more equitable development. From a theoretical perspective, this study contributes to existing knowledge about the development of complex thinking and its sub-competencies, as well as the perceived achievement of professional education students. Although previous studies exist, few are in Latin American realities and with disciplinary differentiation, which gives these results more value, impact, and originality.

### 4.2. Limitations

The present study is limited in several ways. First, the research occurred in a single institution, which makes its results not exhaustive, definitive, or generalizable. Also, not having an initial measurement means not knowing whether the participants effectively developed the competency during their professional education or had already achieved it. Thirdly, it has not been possible to guarantee a large sample of all the careers; however, we can say that the sample is representative, although it is in accordance with the number of graduates, which in some cases are very few. A fourth limitation, which is very relevant to point out, refers to the nature of the scale used, which focuses only on the self-perception of the development of competencies. In this sense, there is a possibility that some students may have biased perceptions that are affecting the results, and therefore, this is a limitation of this type of study. It will be necessary in future studies to compare these results with objective data on the measurement of competencies. Despite these limitations, these results are valuable on an exploratory level, and, as mentioned before, they should be expanded with complementary studies and measurements.

## Figures and Tables

**Table 1:** Characteristics of the population sample.

Discipline	Professional Career	Sample by Professional Career	Sample by Discipline	Percentage of Total
Humanities and Education	Communications	31	31	3.7
Health Sciences	Medicine	55	55	6.6
Social Sciences and Government	International Relations	16	25	3
	Law	9		
Architecture and Design	Architecture	18	98	11.9
	Design	54		
	Digital Art	26		
Engineering	Civil	31	388	46.7
	Industry and systems	57		
	Biotechnology	58		
	Data science and mathematics	8		
	Electronics	3		
	Innovation and development	22		
	Mechanical	24		
	Mechanic administrator	10		
	Biomedical	30		
	Mechatronics	50		
	Chemistry	5		
	Robotics and digital systems	4		
	Computer systems	30		
	Computational technologies	37		
	Digital transformation	2		
	Electronic technologies	6		
	Information technologies	11		
Business	International Business	34	233	28.1
	Business Administration	41		
	Administration and Finance	46		
	Business Creation and Development	10		
	Accounting and Finance	23		
	Business Development	6		
	Organizational development	6		
	Marketing	42		
	Business intelligence	25		
Total			830	100

**Table 2:** Competency and sub-competencies of complex thinking. Mean values and standard deviation by discipline.

		CT	Systemic	Scientific	Critical	Innovative
Overall	Mean	4.30	4.44	4.19	4.29	4.31
	SD	0.46	0.46	0.59	0.52	0.55
HE	Mean	4.35	4.54	4.12	4.39	4.40
	SD	0.44	0.41	0.66	0.46	0.46
SSG	Mean	4.30	4.20	4.25	4.35	4.40
	SD	0.39	0.55	0.44	0.34	0.43
HSc	Mean	4.50	4.49	4.44	4.56	4.52
	SD	0.39	0.45	0.54	0.38	0.42
AAD	Mean	4.22	4.06	4.26	4.36	4.25
	SD	0.52	0.63	0.61	0.52	0.53
ESc	Mean	4.29	4.21	4.28	4.43	4.27
	SD	0.44	0.55	0.54	0.43	0.51
Business	Mean	4.31	4.16	4.34	4.48	4.27
	SD	0.50	0.61	0.54	0.49	0.57

HE = Humanities and Education; SSG = Social Sciences and Government; HSc = Health Sciences; AAD = Architecture, Art and Design; ESc = Engineering and Science.

**Table 3:** Complex thinking and sub-competencies: Analysis of significant differences between mean values in perceived achievement among disciplines (ANOVA).

	Df	Sum Sq	Mean Sq	F Value	Pr (>F)
Systemic Thinking	5	10.73	2.14	6.62	4.67 × 10^−6^ *
Scientific Thinking	5	2.72	0.54	1.79	0.11
Critical Thinking	5	2.13	0.42	2.05	0.06
Innovative Thinking	5	3.79	0.75	2.79	0.01 *
Complex Thinking	5	2.85	0.56	2.63	0.02 *

* *p* < 0.05.

**Table 4:** Mean values and standard deviations of complex thinking in Engineering and Sciences areas.

	Complex Thinking	
Engineering and Sciences	Mean	Sd
Civil	4.38	0.46
Industrial	4.20	0.48
Biotechnology	4.35	0.40
Data Science and Mathematics	4.56	0.42
Electronics	4.40	0.52
Innovation and Development	4.25	0.35
Mechanical	4.27	0.49
Mechanical Administration	4.26	0.32
Biomedical	4.29	0.40
Mechatronics	4.29	0.46
Chemical	4.26	0.61
Robotics and Digital Systems	3.88	0.35
Computer Systems	4.45	0.40
Computational Technologies	4.17	0.51
Digital Transformation	4.66	0.31
Electronic Technologies	4.51	0.44
Information Technologies	4.22	0.33

**Table 5:** Complex thinking analysis of significant differences between mean values in perceived achievement between careers in the School of Engineering and Sciences (ANOVA).

Df	Sum Sq	Mean Sq	F Value	Pr (>F)
18	4.84	0.2687	1.36	0.14

**Table 6:** Mean values and standard deviations of complex thinking in School of Business areas.

	Complex Thinking	
Business	Mean	Sd
International Business	4.33	0.59
Business Administration	4.50	0.41
Administration and Finance	4.32	0.48
Business Creation and Development	4.10	0.37
Accounting and Finance	4.43	0.46
Business Development	4.43	0.34
Organizational Development	4.15	0.35
Marketing	4.19	0.47
Business Intelligence	4.07	0.62

**Table 7:** Complex thinking analysis of significant differences between mean values in the perception of achievement in the careers of the Business School (ANOVA).

Df	Sum Sq	Mean Sq	F Value	Pr (>F)
9	4.98	0.55	2.309	0.01 *

* *p* < 0.05.

**Table 8:** Mean values and standard deviations of complex thinking in Social Sciences and Government.

	Complex Thinking	
Social Sciences and Government	Mean	Sd
Law	4.22	0.35
International Relations	4.36	0.43

**Table 9:** Complex thinking analysis of significant differences between mean values in the perception of achievement between careers in the School of Social Sciences and Government (*t*-test).

t	df	*p*-Value
−0.75	19.65	0.46

**Table 10:** Mean values and standard deviations of complex thinking in Architecture, Art, and Design.

	Complex Thinking	
Architecture, Art, and Design	Mean	Sd
Architecture	4.31	0.52
Digital Art	4.07	0.53
Design	4.27	0.51

**Table 11:** Complex thinking analysis of significant differences between means in perceived achievement between careers in the School of Architecture, Art, and Design (ANOVA).

Df	Sum Sq	Mean Sq	F Value	Pr (>F)
2	0.87	0.43	1.65	0.19

## Data Availability

The data presented in this study are available on request from the corresponding author. The data are not publicly available due to privacy and ethical reasons.
